# Pathogen Detection Using Metagenomic Next-Generation Sequencing of Plasma Samples from Patients with Sepsis in Uganda

**DOI:** 10.1128/spectrum.04312-22

**Published:** 2023-01-10

**Authors:** Brian S. Grundy, Hardik Parikh, Shevin Jacob, Patrick Banura, Chris C. Moore, Jie Liu, Eric R. Houpt

**Affiliations:** a Division of Infectious Diseases and International Health, University of Virginia, Charlottesville, Virginia, USA; b Liverpool School of Tropical Medicine, Liverpool, United Kingdom; c Ministry of Health, National Disease Control Department, Kampala, Uganda; d School of Public Health, Qingdao University, Qingdao, China; University of Maryland School of Medicine

**Keywords:** human immunodeficiency virus, metagenomics, sepsis, sub-Saharan Africa

## Abstract

Metagenomic sequencing is a promising new method for pathogen detection. We aimed to detect pathogens from archived plasma using metagenomic sequencing in a previously well-characterized cohort of 254 predominantly HIV-infected patients with sepsis in Uganda. We used Illumina sequencing and the Chan Zuckerberg ID metagenomics platform to sequence and identify pathogens. On average, each plasma sample yielded 3,404,737 ± 2,201,997 reads (mean ± standard deviation), of which 220,032 ± 416,691 (6.3% ± 8.6%) were identified as nonhuman reads. Using a background model filter, 414 genus-specific pathogen identifications were found in the 254 samples. Nineteen pathogens were previously detected positive by quantitative PCR (qPCR), compared to sequencing, which demonstrated 30.2% sensitivity and 99.5% specificity. Sensitivity was higher for viral pathogens than nonviral pathogens (37% versus 5%). For example, HIV viremia was detected in 69% of samples using qPCR, and sequencing revealed 70% sensitivity and 92% specificity. There were 75 genus-specific potential pathogens identified by sequencing in this cohort, including hepatitis B and Epstein-Barr virus (EBV), among several others. qPCR showed a prevalence of hepatitis B and EBV viremia of 17% and 45%, respectively. In-hospital mortality was associated with a lower qPCR threshold cycle value for EBV (adjusted odds ratio, 0.85; *P* < .001) but not for hepatitis B or HIV. In conclusion, a broad range of potential pathogens were identified by metagenomic sequencing in patients with sepsis in Uganda. Unexpectedly high rates of hepatitis B and EBV viremia were found. Whether these viral infections in HIV patients with sepsis are clinically important requires further study.

**IMPORTANCE** The use of next-generation sequencing (NGS) in blood samples is an emerging technology for clinical microbiology labs. In this work, we performed NGS on plasma samples from a well-characterized cohort, where all samples had been previously tested by PCR for 43 pathogens. Therefore, we could compare sequencing performance against that of PCR and identify clinical correlates. A broad range of potential pathogens were identified by metagenomic sequencing in patients with sepsis in Uganda, particularly viruses, which we confirmed by PCR. In addition to HIV viremia, unexpectedly high rates of hepatitis B and EBV viremia were found, which may have important clinical implications.

## INTRODUCTION

The etiology of sepsis in HIV patients in resource-limited settings is often unknown (1–4). A better understanding of the causative organisms of sepsis can help guide clinicians to select appropriate antimicrobial therapy and avoid the use of unnecessary antibiotics.

Many potential bloodstream infections are often not identified in routine blood cultures (5). For example, there is a high rate of Mycobacterium tuberculosis bloodstream infections in HIV and sepsis (6, 7), but mycobacterial cultures are required for diagnosis. In addition, the diagnosis of several pathogens, including *Leptospira*, *Coxiella*, *Rickettsia*, and Brucella, typically requires serologic testing (8, 9). Many viruses can also cause a sepsis-like picture, and there are several latent viruses that can have additional management implications (10–12). *Plasmodium* and other parasites can also present with severe illness.

A comprehensive diagnostic evaluation for these pathogens, including bacterial cultures, serologies, and antigen testing, is costly and complex. Our previous work using quantitative PCR (qPCR) to detect bacteria, viruses, fungi, and protozoa showed good sensitivity and specificity for pathogen detection from human blood; however, there still remained a large number of specimens with no potential pathogen identified (13, 14). Using a qPCR TaqMan Array card (ThermoFisher), we previously detected a potential pathogen in 72% of a cohort of mostly HIV-infected inpatients with sepsis from Uganda (14). Therefore, many patients still had no pathogens detected, plus not all pathogens detected were causal of sepsis.

Therefore, in this study we aimed to use metagenomic next-generation sequencing (mNGS) for unbiased detection of potential pathogens, including bacteria, fungi, viruses, and parasites, from the available paired plasma samples from this well-characterized cohort. We compared the results with prior qPCR results for whole blood and clinical data.

## RESULTS

There were 671 participants in the full cohort, 336 of whom were previously tested by qPCR and 254 for which we had available plasma. Of the 254 participants, the median (interquartile range [IQR]) age was 35 (28 to 40) years, and 110 (43%) were female. The median (IQR) CD4^+^ count was 60 (13 to 169) cells/μL, the median (IQR) lactate was 3.9 (3.0 to 4.9) mmol/liter, and 65 (27%) died in hospital. The clinical and laboratory data from the 254 plasma samples were not significantly different from the previous qPCR study and the total enrolled cohort (14, 15).

**FIG 1:**
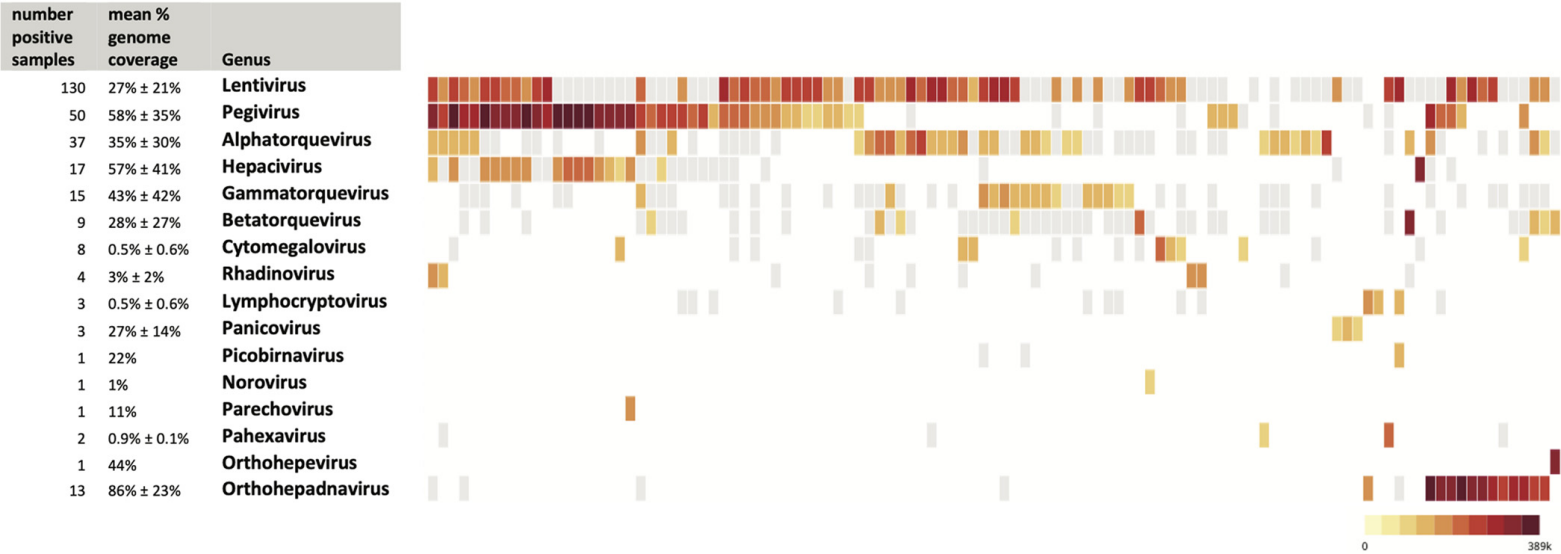
Heatmap of virus genus-specific pathogens identified in plasma samples from patients with sepsis in Uganda. Each column represents a plasma sample from a patient with sepsis in Uganda. Each row represents a virus genus-specific pathogen identified. Colored logarithmic scale represents reads per million (rPM), with darker red representing the highest rPM. Gray boxes represent reads identified for that genus in the sample but not meeting filter threshold criteria. Samples with no viral detection were excluded (*n* = 77), and samples with *Lentivirus* (HIV) as the only virus detected were excluded (*n* = 68).

The plasma samples from Uganda sepsis patients, 4 healthy American plasma samples, and 5 water controls underwent extraction, indexing, pooling, and sequencing. Excluding controls, there was a total of 878,422,177 150-bp paired-end sequencing reads. On average (mean ± SD), each plasma sample had 3,404,737 ± 2,201,997 reads, of which 220,032 ± 416,691 reads (6.3% ± 8.6%) were identified as nonhuman reads. Figure S1 in the supplemental material shows the steps in sequencing and analysis. In total, 414 genus-specific pathogen (295 viral and 119 nonviral) identifications were made in the 254 samples, of which 75 unique genus-specific pathogens (16 viral and 59 nonviral) met threshold criteria (i.e., *Z*-score of >1 above background and >10 reads per million, for both nucleic acids and protein alignment). A total of 207 (81%) samples had at least one pathogen detected, and 139 (55%) samples had at least 2 pathogens detected. The median (IQR) number of pathogens per sample was 2 (1 to 3). Figure 1 highlights the viral pathogens identified in each sample and their relative abundances in number of reads per million. Figure S2A and B show bacterial and eukaryotic pathogens identified, respectively.

For sequencing detections, we evaluated the breadth of sequencing coverage against each pathogen reference NCBI genome. For different pathogens, the percentages of genome coverage were highly variable. Viral detections covered an average of 37% ± 33% of the reference genomes, compared to 16% ± 27% for nonviral pathogens (Fig. 1 and Fig. S2A and B).

Of these genus-level pathogen detections by sequencing, qPCR had been previously performed for 16 pathogens on paired whole-blood samples. For this work, we also performed qPCR for HIV, Epstein-Barr virus (EBV), and hepatitis B virus on the same plasma samples tested by mNGS. Sequencing demonstrated 30.2% sensitivity and 99.5% specificity compared with targeted qPCR (Table 1). Sensitivity of sequencing was higher for viral pathogens than nonviral pathogens (average 37% versus 5%; *P* < 0.001). Detection of HIV using sequencing had a sensitivity and specificity of 70% and 92%, respectively. We performed receiver operating curve (ROC) analysis comparing mNGS reads per million normalized to the genome size for all pathogens tested by qPCR. For viral pathogens, the optimal cutoff was 2.4 reads per million per genome size (megabases). This would yield an overall sensitivity and specificity of 57% and 94% and an area under the curve (AUC) of 0.763. For nonviral pathogens, an optimal cutoff could not be identified, given the low AUC of 0.48 (Fig. S3).

**TABLE 1:** Comparison of qPCR-positive whole-blood samples to sequencing results from plasma samples

Pathogen^*a*^	No. of samples (mean *C_T_*, range) for samples that were positive by qPCR and:		No. of samples negative by qPCR and:		Sequencing vs qPCR sensitivity/specificity
	mNGS positive	mNGS negative	mNGS positive	mNGS negative	
Viral pathogens					
CMV	7 (26.8, 21–31.4)	59 (32.3, 20.9–37)	1	187	11%/99%
Dengue virus	0	5	0	249	0%/100%
EBV*	3 (21, 17.9–23.3)	107 (35.4, 28.6–39.8)	0	144	3%/100%
Hepatitis B virus*	14 (15.2, 10.7–22)	28 (32.4, 22.2–35.3)	0	212	33%/100%
Hepatitis E virus	0	1	1	252	0%/100%
HIV*	124 (28.3, 21.4–37.3)	52 (32.7, 23–39.2)	6	72	70%/92%
Bacterial pathogens					
S. pneumoniae	1	17	1	235	6%/100%
E. coli	0	4	0	250	0%/100%
*Rickettsia* spp.	1	5	0	248	17%/100%
P. aeruginosa	0	1	9	244	0%/96%
K. pneumoniae	0	2	0	252	0%/100%
S. aureus	0	2	2	250	0%/99%
Salmonella spp.	0	2	0	252	0%/100%
*Leptospira* spp.	0	2	0	252	0%/100%
*Coxiella* spp.	0	1	0	253	0%/100%
M. tuberculosis	1	43	0	207	2%/100%
Other pathogens					
*Plasmodium*	2	15	0	234	12%/100%
Cryptococcus	0	6	0	248	0%/100%
*Toxoplasma*	0	2	0	252	0%/100%

a Asterisks denote pathogens detected by qPCR with plasma sample following sequencing.

**FIG 2:**
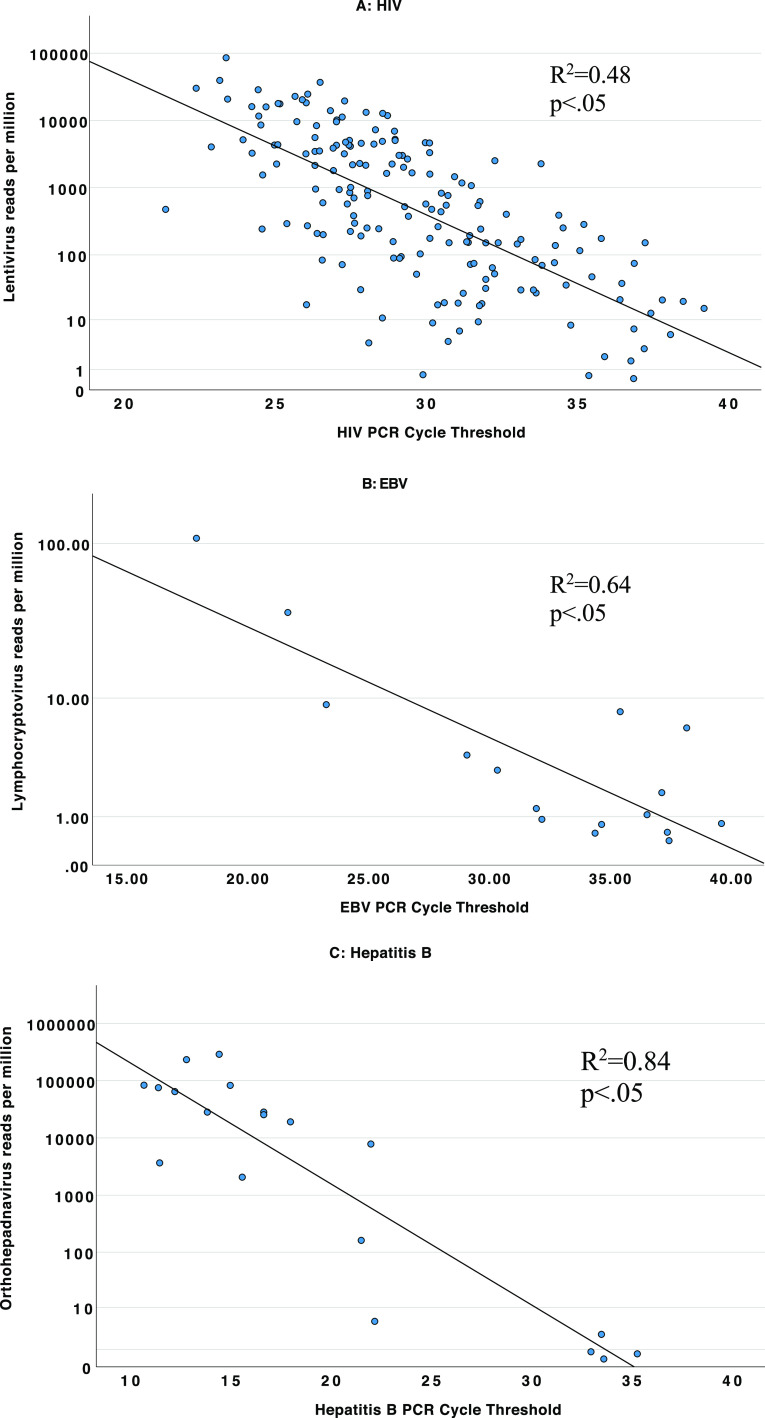
Comparison of qPCR cycle threshold values to sequencing reads per million for HIV, EBV, and hepatitis B virus. (A) Comparison for HIV; (B) comparison for EBV; (C) comparison for hepatitis B virus. Samples that were positive by both PCR and sequencing are shown. All sequencing reads without threshold filters are shown. Note that the *y* axis of each scatterplot is on a logarithmic scale.

Sequencing detected *Lentivirus* (HIV) in 130 samples, *Orthohepadnavirus* (hepatitis B virus) in 14 samples, *Lymphocryptovirus* (Epstein-Barr virus) in 3 samples, and *Cytomegalovirus* in 8 samples. *Hepacivirus* was detected in 17 samples, but only 1 sample aligned to hepatitis C virus species (and was hepatitis C qPCR positive), with the remaining samples aligning to GB virus B species. By qPCR, HIV viremia was detected in 71% of samples, EBV was detected in 45%, and hepatitis B virus was detected in 17% of samples. The quantity of virus detected by qPCR (i.e., threshold cycle [*C_T_*] value) correlated with the number of sequencing reads (Fig. 2).

**FIG 3:**
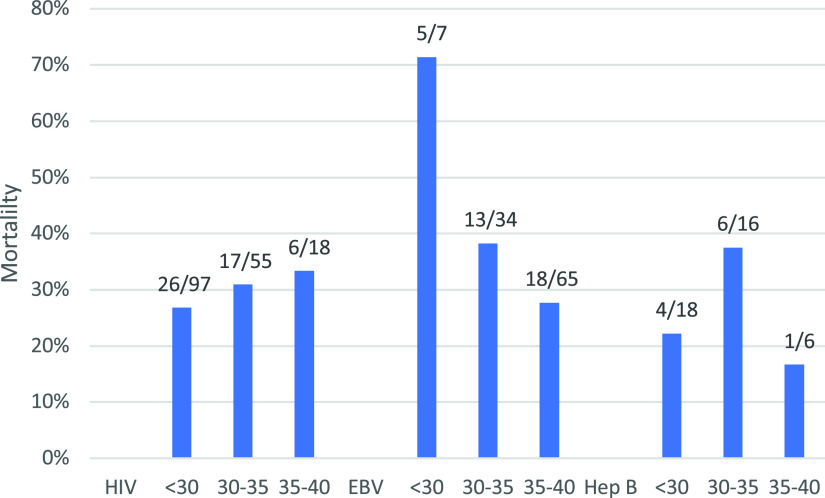
Mortality association with HIV, EBV, and hepatitis B virus by *C_T_* value. The *x* axis shows *C_T_* ranges separated for each pathogen, and the *y* axis shows the in-hospital mortality of each pathogen by *C_T_* value. The fractions above each bar represent the numbers of deaths within each *C_T_* range as the numerator and the number of samples in that *C_T_* range as the denominator.

Mortality was associated with a lower *C_T_* value for EBV (adjusted odds ratio of 0.85, *P* < 0.001) but not for hepatitis B virus or HIV (Fig. 3). There was no significant correlation between total sequencing reads, pathogen reads, or pathogen-specific reads with regard to mortality or lactate concentration.

## DISCUSSION

In this study, we demonstrated the ability of metagenomic next-generation sequencing from plasma samples to detect potential etiologic pathogens contributing to sepsis in Uganda. We found that the mNGS method was relatively insensitive for many bacterial pathogens but was moderately successful for many viruses. We successfully identified HIV in the majority of samples and detected new pathogens, including EBV and hepatitis B virus, among several others that may be important.

On average, each plasma sample had 3,404,737 ± 2,201,997 reads, of which 220,032 ± 416,691 reads (6.3% ± 8.6%) were identified as nonhuman potential pathogen reads. This depth was similar to other studies using mNGS sequencing from plasma or serum for pathogen detection (16), and the percentage of nonhost reads from plasma samples was higher than that in some other studies (16, 17). This could have been contributed by the use of human rRNA depletion in the cDNA library preparation, the extreme illness of our cohort, and/or the high HIV rate, since HIV was the leading pathogen identified.

Background filtering allowed for an unbiased way to remove potential contaminant reads from the data, as highlighted in Fig. 1, without which nearly every sample had some level of pathogen reads including in healthy control plasma samples and water samples (for instance, these samples revealed bacterial reads such as Escherichia and Klebsiella, among others). If a pathogen was detected in our water and healthy controls, such as several bacterial species, they were filtered out, even if they were potentially a true pathogen in the samples. While the filtering thresholds were somewhat arbitrary, they could be adjusted. We aimed to keep high filter thresholds to maintain high specificity at the expense of sensitivity. The appropriate filter threshold was difficult to determine and can differ for different pathogens based on several factors, including size of pathogen genome, level of pathogen burden in serum, and depth of sequencing (18–20).

The sensitivity of mNGS compared to targeted qPCR was low, at 30% overall, and even lower for bacteria at 5%. This was not surprising to us and can likely explained by several factors, including the read depth, limited sample volume, age of the samples (10+ years), different sample types (sequencing on plasma versus qPCR on whole blood), stringency of the background filtering thresholds used, and the potential presence of inhibitory substances to sequencing in human plasma, which have all been previously described (21–23). Several studies have shown similarly variable sensitivity and specificity of mNGS versus PCR (24–27). Additionally, our extraction and library preparation methods aimed to sequence both RNA and DNA viruses, bacteria, and fungi by using RNA sequencing, which may have preferentially favored some pathogens over others, as previously described (27–29).

We evaluated the percent reference genome coverage of pathogens in each sample to better characterize identified pathogens and increase specificity, by requiring that the reads mapped to diverse sites and were less likely to have been a single contaminant region or genome database anomaly. This approach, however, would favor pathogens with smaller reference genomes or reference accessions that use incomplete genomes. This likely contributed to the higher viral genome coverage.

A total of 75 genus-specific pathogens were identified that passed the filter threshold criteria. The most prevalent pathogen, not surprisingly, was HIV (*Lentivirus*), which was detected by this method in 130 samples. HIV was detected by qPCR in 176 samples. Other prevalent pathogens included pegivirus, seen in 50 samples, which is highly prevalent globally but not known to be of clinical importance (30). The pegivirus prevalence here was similar to that noted in an HIV-infected cohort in Ghana (20% versus 16%, respectively) (10). Several of the torque viruses were highly prevalent in this cohort, which was not surprising as these viruses are ubiquitous human viruses approaching possibly 100% prevalence in the human population, with no known clinical significance (31). Additionally, hepatitis B (*Orthohepadnavirus*) was seen in 13 samples, which can have significant implications if acute infection contributing to sepsis or the chronic management of this virus. *Hepacivirus* (the genus of hepatitis C virus) was detected by sequencing in 17 samples, but on analysis of species-level alignments in only one sample was hepatitis C detected (and confirmed by qPCR), with the remaining samples aligning to the closely related GB virus B, of which viremia is known to be highly prevalent but not known to cause human disease (32). *Rhadinovirus* (human herpesvirus 8 or Kaposi’s sarcoma-associated herpesvirus) was detected in 4 samples; this genus is often associated with HIV and has important clinical implications of potential malignancy (33). Several of the other genera identified, including many bacteria and fungi (as highlighted in Fig. S2A and B), are atypical for human bloodstream infections and may have represented environmental contamination, commensal organisms, or etiologic pathogens in this highly immunocompromised HIV-infected population.

Sequencing of HIV was evaluated in this cohort, given its high prevalence and importance in contributing to the risk of opportunistic and atypical infections in sepsis. The sensitivity and specificity of mNGS for the detection of HIV compared to qPCR were 70% and 92%, respectively. We suspect some reduced sensitivity of molecular diagnostics for HIV in this cohort, given 23% of HIV-positive patients were known to be taking antiretroviral therapy and a portion may have been virologically suppressed (15). Despite this possibility, the cohort had a low median CD4 level, suggestive of poor virological control, but qPCR and mNGS results did not correlate with CD4 count. Mortality was not associated with qPCR for HIV, which highlighted other potential pathogens as the primary cause of this cohort’s acute illness, sepsis, and mortality.

EBV (*Lymphocryptovirus*) was only detected in 3 samples using this mNGS method, but when tested by qPCR, 45% of samples were found to be positive. These findings presumably reflected the insensitivity of mNGS compared to targeted qPCR. The high prevalence of EBV viremia by qPCR in this cohort was higher than we anticipated and higher than reported in a similar study of febrile illness in Kenya (29%) (34). This high-level viremia likely reflected the severity of illness in this cohort and the activation of EBV-infected immune cells (35). It is possible that the association between high EBV viremia and mortality may be clinically relevant or related to potential EBV-related malignancy; however, we were unable to evaluate this explicitly in this cohort.

Hepatitis B (*Orthohepadnavirus*) viremia was also unexpectedly found in 17% of samples by qPCR. This was higher than expected, given the WHO estimate of HBsAg seroprevalence of 6.1% in sub-Saharan Africa and a reported 10.9% viremia in symptomatic HIV-infected patients in Ghana (10, 36). While again not likely to be significantly contributing to sepsis or associated with mortality in this cohort, the high prevalence of viremia has implications for the acute and chronic management of hepatitis B and the frequent coinfection with HIV.

This study had several limitations. The plasma samples had been cryopreserved for about 12 years prior to sequencing, while the comparison whole-blood samples had been cryopreserved for about 9 years prior to PCR (14, 37); therefore, the prolonged storage could have affected detection yield. Similarly, the extraction and sequencing methods used and the background model from healthy human and water control samples could have variably impacted the pathogens detected. The turnaround time and cost of both qPCR and mNGS are a limitation for the routine use of these molecular diagnostics in clinical care. The 48-plex qPCR using the TaqMan Array card had an approximate turnaround time of a few hours at about $100 per sample, while mNGS required a few days at a cost of about $500 per sample (37). While there are several available bioinformatics methods, we used the Chan Zuckerberg ID bioinformatics pipeline in this study; however, when we examined the results using an in-house method, we found similar results. Specifically, we evaluated our results using a different bioinformatics pipeline with K-mer-based species identification and found similar results (Table S1). Notably, all such analyses are dependent on the quality of the NCBI reference library used. The bioinformatics methods used may also likely identify pathogens with smaller genomes and are limited by the depth of sequencing. Analyses of clinical correlations, such as lactate concentrations, and mortality with individual pathogens were limited by the relatively small number of specific pathogen detections in this cohort.

In summary, in a predominantly HIV-infected population with sepsis, we were able to use mNGS to detect pathogens in plasma and compare these results to prior qPCR, culture, and clinical data. While relatively insensitive compared to qPCR, our methods were able to detect several new infections, especially viral infections, that could be further assessed by qPCR. mNGS may serve as a useful adjunctive tool to identify new pathogens not detected by other methods.

## MATERIALS AND METHODS

### Sample collection.

Patient enrollment and specimen collection have been described previously (15). Individuals were ≥18 years of age with sepsis and were enrolled in a fluid resuscitation study from the medical casualty units of Mulago National Referral Hospital and Masaka Regional Referral Hospital in Uganda from May 2008 to May 2009. Baseline demographic, clinical, and routine lab data were obtained. Briefly, among the 336 patients tested previously by PCR, we tested all available cryopreserved plasma specimens (*n* = 254). We also collected blood from 4 healthy American volunteers as negative controls. All plasma samples were cryopreserved at −80°C until they were tested in 2021. qPCR detection for 43 targets from the paired whole blood (collected at the same time as the plasma samples) has been previously described (14).

### Ethics statement.

Written informed consent was obtained from each patient or a surrogate if the patient could not provide consent. All work was approved by the University of Virginia Institutional Review Board.

### Library preparation and sequencing.

Using 1 mL of plasma from each patient, total nucleic acid was extracted using the QIAamp circulating nucleic acid kit (Qiagen) following the kit protocol into a 30-μL elution volume. cDNA was created from 6 μL of elution mixture using the REPLI-g WTA single-cell kit (Qiagen) according to the manufacturer’s instructions, with the addition of human rRNA removal using the QIAseq FastSelect −rRNA HMR kit (Qiagen). In total, 263 samples (254 plasma samples, 4 healthy human plasma samples, and 5 water controls) were indexed and pooled using the Nextera DNA Flex library prep kit and run on an Illumina Hiseq 2500 system to obtain 150-bp paired-end reads. Confirmatory qPCRs were performed on the extracted plasma for HIV, EBV, and hepatitis B virus using previously described primers and probes (13, 38–40).

### Data processing.

Microbial pathogens were identified from raw sequencing reads using Chan Zuckerberg ID (v6.6), a cloud-based, open source bioinformatics platform. In brief, initial host read filtering was performed using spliced transcripts alignment to a reference (STAR) algorithm, followed by removal of duplicate or low-quality and low-complexity sequences. Next, reads were aligned to the host genome of interest using bowtie2 to remove any remaining host reads. The nonhuman reads were then aligned to the NCBI nucleotide and protein database (NCBI index date, 20 April 2020), using GSNAPL and RAPSearch, respectively.

All Chan Zuckerberg ID scripts and user instructions are available at https://github.com/chanzuckerberg/idseq-dag, and the graphical user interface web application for sample upload is available at https://github.com/chanzuckerberg/idseq-web. Further user guides and explanations can be found at https://chanzuckerberg.zendesk.com/hc/en-us. Using suggested threshold filters from CZidseq, a *Z*-score was calculated for both nucleic acid and protein alignments for each genus relative to a background of nontemplate (water only) and healthy human plasma controls, whereby a genus with a *Z*-score of >1 and with >10 reads per million and for both nucleic acids and protein alignment in a sample were considered positive. For further specificity in the detection of new pathogens, the percent coverage for each pathogen to the reference NCBI accession was evaluated. Based on the sequencing results, we selected HIV, HBV, and EBV to further evaluate the results by targeted qPCR. qPCR was considered positive for detection if the *C_T_* was <40.

### Statistics.

We calculated sensitivity and specificity for sequencing results compared to previous qPCR results. We also performed ROC analysis by comparing pathogen reads per million without any background thresholds, divided by each reference NCBI genome size against PCR. We evaluated relationships between sequencing and qPCR results with in-hospital mortality, CD4, HIV status, and lactate concentration with logistic regression and adjusted for age and sex. *P* values were 2-tailed, and values of <0.05 were considered significant (SPSS, version 28, IBM Corp, Armonk, NY).

### Data availability.

Sequencing data from this study are publicly available under the accession number PRJNA898830. Data that were processed through the Chan Zuckerberg ID pipeline are publicly available at https://czid.org/ under the project title “Uganda Sepsis Metagenomics Nextseq.”
