# Supplementary material for: Pathogen Detection Using Metagenomic Next-Generation Sequencing of Plasma Samples from Patients with Sepsis in Uganda
Source: Microbiol Spectr. 2023 Jan 10;11(1):e04312-22. doi: 10.1128/spectrum.04312-22 (PMC9927450; doi:10.1128/spectrum.04312-22)
Supplement: Supplemental file 1 — Supplemental material. Download spectrum.04312-22-s0001.pdf, PDF file, 0.7 MB [file spectrum.04312-22-s0001.pdf]

Supplemental Figure 1: Workflow of RNA extraction, sequencing, bioinformatics and key findings

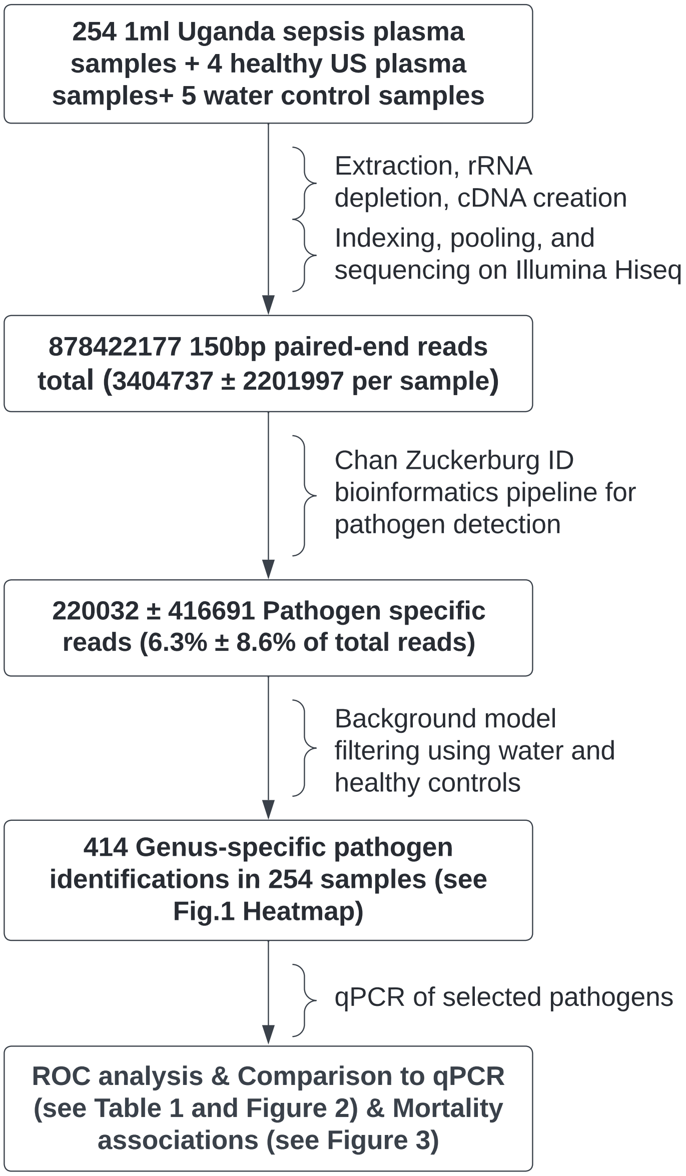

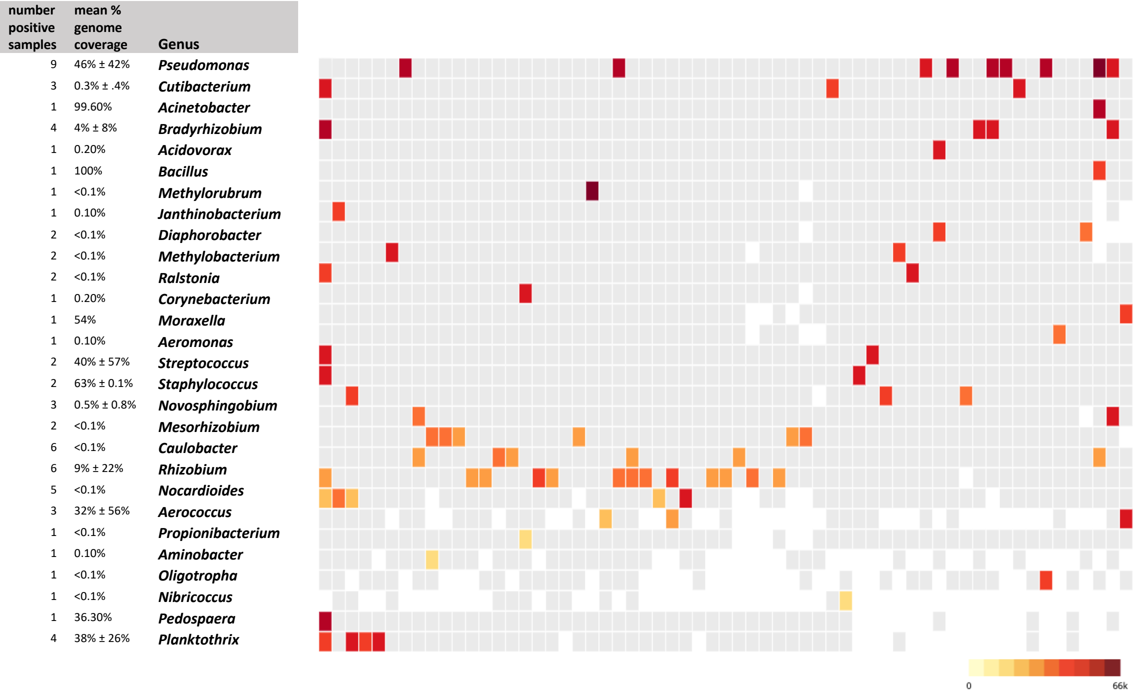

Figure 2A – Heatmap of Bacteria Genus-specific pathogens identified in plasma samples from patients with sepsis in Uganda. Each column represents a plasma sample from a patient with sepsis in Uganda. Each row represents a Bacteria Genus-specific pathogen identified. Colored logarithmic scale represents reads per million (rPM) with darker red representing the highest rPM. Grayed boxes represent reads identified for that genus in the sample but not meeting filter threshold criteria. Samples with no bacterial detection are excluded (n=193).

number positive samples	mean % genome coverage	Genus
1	93%	***Phaffia***
2	10% ± 0.2%	***Phaeodactylum***
1	0.4%	***Haslea***
2	10% ± 14%	***Trypanosoma***
1	22%	***Navicula***
1	<0.1%	***Alternaria***
1	28%	***Euglena***
1	15%	***Tetrahymena***
2	5% ± 7%	***Chlorella***
1	18%	***Panicum***
1	43%	***Exophiala***
1	0.3%	***Aegilops***
1	17%	***Rhizopus***
1	<0.1%	***Temnothorax***
1	12%	***Thrichoderma***
1	57%	***Euglypha***
1	9%	***Metarhizium***
2	24% ± 18%	***Aureobasidium***
1	39%	***Olea***
1	<0.1%	***Rhodotorula***
1	<0.1%	***Musa***
1	27%	***Philodina***
1	4%	***Setaria***
3	17% ± 8%	***Loa***
1	28%	***Brugia***
2	0.1% ± 0.1%	***Plasmodium***
1	2%	***Hordeum***
2	<0.1%	***Triticum***
3	9% ± 15%	***Oryza***
2	18% ± 26%	***Zea***
10	8% ± 12%	***Penicillium***

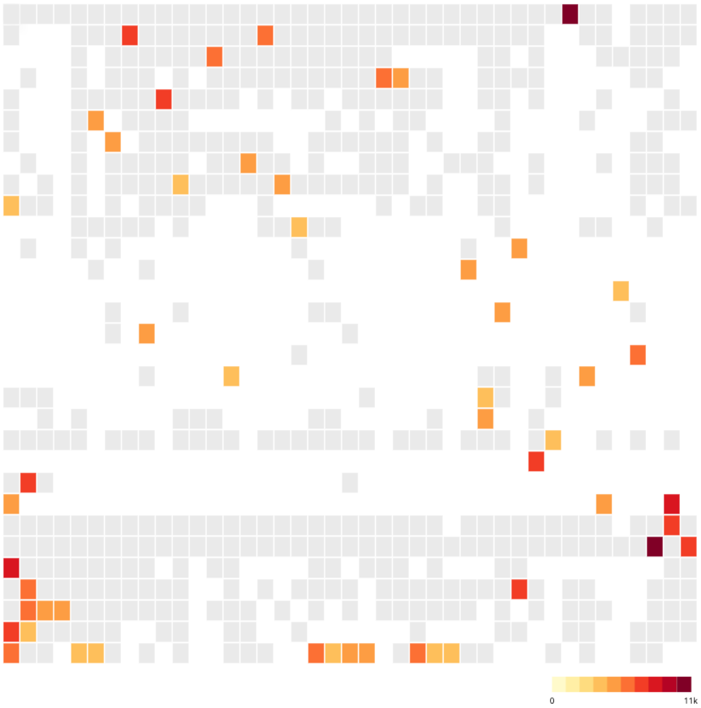

Figure 2B – Heatmap of Eukaryotic Genus-specific pathogens identified in plasma samples from patients with sepsis in Uganda. Each column represents a plasma sample from a patient with sepsis in Uganda. Each row represents a Eukaryotic Genus-specific pathogen identified. Colored logarithmic scale represents reads per million (rPM) with darker red representing the highest rPM. Grayed boxes represent reads identified for that genus in the sample but not meeting filter threshold criteria. Samples with no eukaryotic detection are excluded (n=213).

Figure 3: Receiver operator curves for viral and non-viral detection by reads per million by genome size (Mb) compared to qPCR positivity

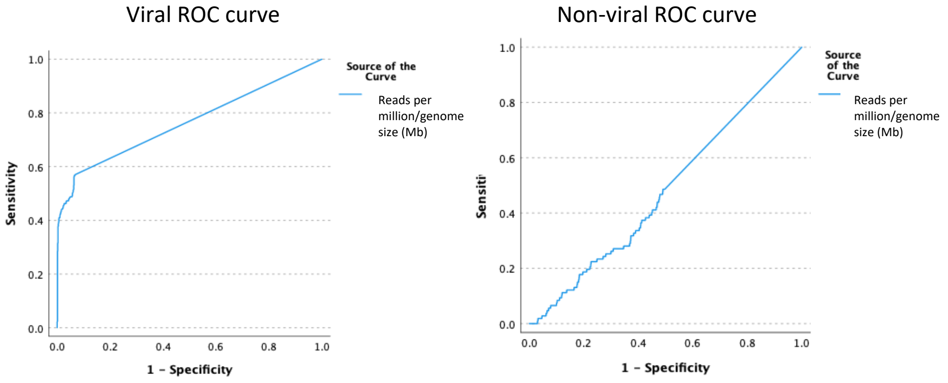

Supplemental Table 1: Species with greater than 1% genome coverage detected using K-mer based bioinformatics method.

Species	Number of samples
Human immunodeficiency virus 1	66
Hepatitis B virus	12
Torque teno virus 5	5
Toxoplasma gondii	5
Human betaherpesvirus 5 (CMV)	4
Human gammaherpesvirus 8 (HHV-8)	4
Pseudomonas azotoformans	3
Streptococcus pneumoniae	3
Torque teno virus 22	3
Torque teno virus	2
Torque teno virus 13	2
Torque teno virus 15	2
Torque teno virus 16	2
Torque teno virus 18	2
Torque teno virus 27	2
Acinetobacter junii	1
Actinobacillus sp. GY-402	1
Aerococcus urinaeequi	1
Aerococcus viridans	1
Aeromonas hydrophila	1
Bermuda grass latent virus	1
Capuchin monkey hepatitis B virus	1
Escherichia albertii	1
Human gammaherpesvirus 4 (EBV)	1
Pegivirus C	1
Plasmodium falciparum	1
Pseudomonas sp. Y39-6	1
Sarcina sp. JB2	1
Streptococcus mitis	1
Streptococcus sp. oral taxon 061	1
Streptococcus suis	1
Torque teno mini virus 11	1
Torque teno virus 19	1
Torque teno virus 29	1
Torque teno virus 9	1
